# Manipulating the Amount and Structure of the Organic Matrix Affects the Water Compartments of Human Cortical Bone

**DOI:** 10.1002/jbm4.10135

**Published:** 2019-01-28

**Authors:** Jeffry S Nyman, Sasidhar Uppuganti, Mustafa Unal, Calen J Leverant, Saahit Adabala, Mathilde Granke, Paul Voziyan, Mark D Does

**Affiliations:** ^1^ Department of Orthopaedic Surgery and Rehabilitation Vanderbilt University Medical Center Nashville TN USA; ^2^ Department of Biomedical Engineering Vanderbilt University Nashville TN USA; ^3^ Department of Veterans Affairs Tennessee Valley Healthcare System Nashville TN USA; ^4^ Department of Chemical and Biomolecular Engineering Vanderbilt University Nashville TN USA; ^5^ Department of Medicine Division of Nephrology Vanderbilt University Medical Center Nashville TN USA; ^6^ Department of Radiology and Radiological Sciences Vanderbilt University Medical Center Nashville TN USA; ^7^ Department of Electrical Engineering Vanderbilt University Nashville TN USA

**Keywords:** ^1^H NUCLEAR MAGNETIC RESONANCE, RAMAN SPECTROSCOPY, MATRIX BOUND WATER, ADVANCED GLYCATION END PRODUCTS, TYPE 1 COLLAGEN, BONE QUALITY, SECONDARY STRUCTURE

## Abstract

Being predictors of the mechanical properties of human cortical bone, bound and pore water measurements by magnetic resonance (MR) imaging are being developed for the clinical assessment of fracture risk. While pore water is a surrogate of cortical bone porosity, the determinants of bound water are unknown. Manipulation of organic matrix properties by oxidative deproteinization, thermal denaturation, or nonenzymatic glycation lowers bone toughness. Because bound water contributes to bone toughness, we hypothesized that each of these matrix manipulations affect bound water fraction (V_bw_/V_bone_). Immersing cadaveric bone samples in sodium hypochlorite (NaClO) for 96 hours did not affect tissue mineral density or cortical porosity, but rather decreased V_bw_/V_bone_ and increased short‐T_2_ pore water signals as determined by ^1^H nuclear MR relaxometry (^1^H NMR). Moreover, the post treatment V_bw_/V_bone_ linearly correlated with the remaining weight fraction of the organic matrix. Heating bone samples at 110°C, 120°C, 130°C, and then 140°C (∼24 hours per temperature and rehydration for ∼24 hours before ^1^H NMR analysis) did not affect V_bw_/V_bone_. After subsequently heating them at 200°C, V_bw_/V_bone_ increased. Boiling bone samples followed by heating at 110°C, 120°C, and then 130°C in water under pressure (8 hours per temperature) had a similar effect on V_bw_/V_bone_. Raman spectroscopy analysis confirmed that the increase in V_bw_/V_bone_ coincided with an increase in an Amide I subpeak ratio that is sensitive to changes in the helical structure of collagen I. Glycation of bone by ribose for 4 weeks, but not in glucose for 16 weeks, decreased V_bw_/V_bone_, although the effect was less pronounced than that of oxidative deproteinization or thermal denaturation. We propose that MR measurements of bound water reflect the amount of bone organic matrix and can be modulated by collagen I helicity and by sugar‐derived post translational modifications of the matrix. © 2019 The Authors. *JBMR Plus* published by Wiley Periodicals, Inc. on behalf of American Society for Bone and Mineral Research.

## Introduction

Comprising approximately 20% of the volume of bone, water exists in the pores of the vascular‐lacunar‐canalicular network and bound to the matrix via hydrogen bonding and electrostatic attractions.^1^ As determined by magnetic resonance (MR) techniques, the pore water volume fraction is directly proportional to intracortical porosity of bone.^2^, ^3^ As such, the level of MR‐derived pore water signals is negatively associated with the strength of cortical bone at the apparent level (independent of macrostructure but not microstructure).^4^, ^5^ The mechanical properties of bone have long been known to depend on the degree of hydration,^6^ and bound water specifically confers plasticity—the ability to deform after yielding—to bone.^7^ Although water is known to provide stability to collagen,^8^, ^9^ the matrix‐related factors dictating the volume fraction of bound water in bone have yet to be identified.

The early studies of bone involving gravimetric measurements of water content (wet mass minus dry mass after thermal dehydration) found that water content decreased with skeletal maturation^(10)^ and was inversely proportional to ash fraction,^11^ leading to the concept that the accumulation of mineral within osteoid displaces bound water.^12^ Not surprisingly then, we observed that MR‐derived bound water fraction (volume of H_2_O per apparent volume of bone) of femurs or femur midshafts was negatively correlated with tissue mineral density (TMD), as determined by micro‐computed tomography (µCT), in two rodent models of aging.^1^ Accompanying the age‐related increase in TMD and decrease in bound water fraction was an increase in pentosidine, a nonenzymatic, glycation‐mediated collagen cross‐link. Thus, bound water fraction depends on the degree of mineralization, but other matrix‐related factors could also contribute to the volume fraction of bound water in bone.

Unlike rodent cortical bone, human bone experiences extensive turnover or remodeling throughout life and so the overall degree of mineralization does not increase with age after skeletal maturation,^13^ albeit there may be a shift toward higher fraction of hypermineralization with aging.^14^ Also, in human cortical bone, bound water fraction decreases with advanced aging without an increase in TMD.^1^, ^7^ There are potentially several age‐related changes to the organic matrix of bone that could be related to the decrease in bound water fraction: 1) an increase in advanced glycation end products (AGEs);^15^, ^16^ 2) a decrease in the thermal stability of collagen I fibrils^17^, ^18^ (ie, less stable collagen despite increases in AGE cross‐links); 3) a decrease in the maximum rate of contraction of demineralized bone during hydrothermal isometric shrinkage^19^ (ie, reduced collagen integrity related to how well the collagen I network is interconnected); 4) a decrease in the amount of glycosaminoglycans^20^ (ie, reduction in natural compounds that attract water); and 5) an increase in type B carbonate substitutions.^21^ Therefore, the volume fraction of bound water in bone is likely to have multiple interrelated determinants.

As determined by ^1^H nuclear magnetic resonance (^1^H NMR) relaxometry and related MR imaging (MRI) techniques, we^3^, ^5^, ^22^ and others^23^ have found that the amount of matrix‐bound water directly correlates to such mechanical properties of cortical bone as strength, toughness, and fracture toughness, and does so independently of pore water.^3^, ^5^, ^22^ Currently, ultra‐short echo‐time (UTE) MRI techniques are being developed to quantify bound and pore water concentrations in humans for potential improvement in the prediction of a patient's fracture risk.^24^ The proton signals of bound water (T_2_ ≃ 0.1 ms to 1 ms) are known to exchange magnetization with proton signals having a shorter relaxation time (T_2_ ≃ 0.02 ms to 0.1 ms),^25^ which indicates that bound water likely interacts with amino acid residues of proteins (eg, α_1_(I) and α_2_(I) chains of type 1 collagen or collagen I). Nonetheless, the degree to which the NMR‐derived bound water signals depend on collagen I structure and AGE accumulation is unclear.

Because thermal denaturation of collagen I,^26^ partial deproteinization of the bone matrix,^27^ and glycation to increase matrix AGE levels^28^ all can decrease bone toughness or failure strain, we hypothesized that these ex vivo treatments would also decrease bound water fraction as determined by ^1^H NMR relaxometry.

## Materials and Methods

### Bone sample preparation

Fresh‐frozen cadaveric femurs were obtained from two tissue banks (NDRI, Philadelphia, PA, USA, and MTF Biologics, Edison, NJ, USA), and femur midshafts were obtained from the Vanderbilt Donor Program (Nashville, TN, USA). The bones were stored at −80°C unless being cut to prepare specimens. Using a circular, diamond‐embedded saw with irrigation (Model 660, South Bay Tecnologies, Inc., Torrance, CA, USA), strips of cortical bone (∼2.5 to ∼3.4 mm in thickness and ∼70 mm in length) were extracted from the medial or lateral quadrant of the diaphyseal cross sections (specimens were from the same quadrant within each experiment). The periosteal and endosteal surfaces were further removed with an end‐mill (∼4.5 mm in width), also under irrigation, and stored immersed in phosphate‐buffered saline (PBS; pH 7.4, P‐3813, Sigma‐Aldrich Co., St. Louis, MO, USA) at −20°C. One longitudinal surface (∼4.5 mm × ∼70 mm) of each parallelepiped was ground using a 400 CS Micro Grinder System (EXAKT Technologies, Inc., Oklahoma City, OK, USA) with irrigation going from coarse silica carbide paper (270 mm K1200, EXAKT Technologies, Inc.) to fine silica carbide paper (300 mm K4000, EXAKT Technologies, Inc.) and then polished using a Vibromet 2 (Buehler USA Inc., Lake Bluff, IL, USA) with a synthetic cloth (12″ MASTERTEX PSA, p/n 40‐7742, Buehler USA Inc.) coated in an alumina oxide solution (0.05 µm MasterPrep Polishing suspension, Buehler USA Inc.). Before manipulation, samples were cut from the strips using the same low‐speed circular saw to provide nominal dimensions of ≈5 mm × 5 mm × 2 mm, except for one experiment in which the width was ≈8 mm (baking). Measurements of wet mass and submerged mass (SI‐215D electronic balance, Denver Instrument, Bohemia, NY, USA) were used to determine apparent bone volume by Archimedes’ principle.

### Deproteinization with sodium hypochlorite

To reduce the amount of organic matrix without affecting the mineral content, bone samples were placed in 2 mL of reagent‐grade NaOCl (425044, Sigma‐Aldrich) and left on a shaker plate at room temperature for 96 hours (batch 1: 4 males, age range: 38 to 97 years, 74.5 ± 25.4 years, and 6 females, age range: 42 to 101 years, 73.7 ± 23.7 years; batch 2: 6 males, age range: 46 to 91 years, 72.8 ± 17.9 years and 4 females, age range: 32 to 89 years, 58.7 ± 23.5 years; Supplemental Table S1).

### Thermal denaturation

To denature the collagen, samples were first heated in an oven (model VO914A, Thermo‐Fisher Scientific, Waltham, MA, USA) as follows: baked at 110°C, 120°C, 130°C, 140°C, and 200°C for 24 hours per temperature in successive order (10 males, age range: 21 to 98 years, 63.6 ± 30.4 years; Supplemental Table S1). The specimens were rehydrated at 4°C in PBS for 24 hours and weighed before analysis. To confirm the effect of thermal denaturation on bound water without multiple cycles of dehydration and rehydration (hence, cycles of collagen contraction and expansion, respectively), we also boiled additional bone samples for 8 hours and then sealed them in a Pyrex tube with 10 mL of water and heated them in the oven at 110°C, 120°C, and 130°C for 8 hours per temperature in successive order (10 males, age range: 25 to 91 years, 61.9 ± 23.8 years and 10 females, age range: 29 to 101 years, 66.1 ± 26.7 years; Supplemental Table S1).

### Glycation with ribose and glucose

To increase AGE content, 3 bone samples from each donor (5 males, age range: 46 to 60 years, 54.8 ± 5.5 years, and 5 females, age range: 53 to 58 years, 55.4 ± 2.1 years; Supplemental Table S1) were incubated in 100 mM sodium phosphate buffer for 4 weeks without sugar or with 0.1 M or 0.5 M D‐ribose (R7500, Sigma‐Aldrich) at 37°C. An additional 3 bone samples from the same donors were incubated as described above but also had 50 mM pyridoxamine added. For glucose incubations, 3 bone samples from each donor (5 males, age range: 46 to 60 years, 54.8 ± 5.5 years, and 5 females, age range: 47 to 60 years, 54.2 ± 4.9 years; Supplemental Table S1) were incubated either without sugar or with 0.1 M or 0.5 M D‐glucose (G8270, Sigma‐Aldrich) as described above for a total period of 16 weeks with analysis at 4 weeks, 8 weeks, and 16 weeks. Sodium azide (final concentration of 0.02%) was added to all the buffered solutions to prevent bacterial growth. The pH range for all groups was maintained between 7.0 and 7.5 during incubation by addition of small amounts of 10 N NaOH. After incubation, all bone specimens were rinsed with PBS, placed in this buffer, and stored at −20°C until analyzed.

### ^1^H NMR

Before, after, or between different manipulation steps, bound water and pore water signals were acquired by Carr‐Purcell‐Meiboom‐Gill (CPMG) measurements at a field strength of 4.7 T (horizontal bore with a diameter of 11.8 cm, Varian Medical Systems, Santa Clara, CA, USA) following our previously published methods.^5^, ^25^ Briefly, after thawing to room temperature, each hydrated bone sample and a reference microsphere of water (T_2_ ≃ 2 s) were sealed in a 10 mm NMR tube (513‐1PP‐7, Wilmad LabGlass, Vineland, NJ, USA) and then placed in a custom‐built, low‐proton, loop‐gap‐style radio‐frequency (RF) coil that provided 90°/180° RF pulses of ∼5 µs/∼10 µs duration.^29^ Collecting 10,000 echoes at 100 µs spacing, signals were fitted with multiple exponential decay functions to generate the separate pools of proton signals as a T_2_ spectrum. The integrated areas of the bound water pool and pore water pool were converted to volume of H_2_O using the integrated area of the reference volume of H_2_O. These measurements were then normalized to the apparent volume of the bone specimen.

### Raman spectroscopy

To determine whether each heating protocol denatured collagen or whether there were differences in the secondary structure of collagen I among the sugar groups, Raman spectra were collected from the polished surface of each specimen before heating (baseline) and then after heating at each temperature or after incubation in sodium phosphate buffer, respectively. Described in detail elsewhere,^21^ Raman spectra were acquired at four sites (two interstitial and two osteonal) using one of two InVia confocal Raman microscopes (Renishaw, Gloucestershire, UK) as follows: 1) 785 nm laser diode with spot focus, 3 accumulations of a 10‐second exposure, and a 50 µm slit (baking experiment); 2) 830 nm laser diode with line focus, 6 accumulations of a 20‐second exposure, and 55 µm slit (boiling and pressure‐heating experiment); and 3) 785 nm laser diode with line focus, 6 accumulations of 20‐second exposure, and 55 µm slit (sugar experiments). In each instance, we used a 50× objective (NA = 0.75) and a holographic grating to a thermoelectrically cooled, deep‐depleted, charge‐coupled diode (CCD) that provided 1 cm^−1^ spectral resolution. After the samples were baked at 200°C, they were a dark reddish color causing a strong fluorescence that saturated the CCD and obscured the Raman signal of bone. To minimize the strong fluorescence background from these samples, we used a photobleaching process. Before Raman data acquisition described above, these samples were irradiated by the laser at 35 mW power for 15 minutes via photobleaching option of the Raman microscope. This reduced the fluorescence background by 30% to 40% of the initial value, thereby enabling the acquisition of the Raman signal of the bone samples baked at 200°C. All spectra were processed to determine Raman measurements of bone matrix composition following our published methods.^30^

### Gravimetric measurements

To determine the amount of organic matrix that remained after deproteinization in the sodium hypochlorite experiment, bone samples were weighed after vacuum drying (470 mm Hg) at room temperature for 24 hours, after oven drying at 200°C for 24 hours, and after ashing at 600°C for 24 hours in a furnace (Thermolyne F‐A10525P, Sybron Inc., Dubuque, IA, USA). The remaining organic fraction was air‐dry mass minus the ash mass normalized to air‐dry mass expressed as percentage.

### Micro‐computed tomography

To confirm that deproteinization protocol did not affect mineral content, bone samples were imaged by µCT as previously described.^3^ Briefly, the long axis of each specimen was aligned with scanning axis of a µCT50 (Scanco Medical AG, Brüttisellen, Switzerland), and an image stack was acquired at an isotropic voxel size of 5 µm with the following scan parameters: peak tube voltage of 90 kVp; tube current of 200 µA; 1000 projections per 360‐degree rotation, integration time of 400 ms, and a beam hardening correction factor for a hydroxyapatite (HA) phantom calibration (ie, 1200 mgHA/cm^3^). After reconstruction, the µCT image stack was loaded in the Scanco evaluation program (v6.6) and tight‐fitting contours were drawn around the bone sample within each slice to define the region of interest for evaluation. Scanco evaluation scripts were run to calculate: 1) the total cross‐sectional area (TA) and TMD using a Gaussian filter (sigma = 1.8 and support = 3.0) to suppress image noise and a threshold between 595.4 and 3000 mgHA/cm^3^ to select the bone voxels and 2) cortical porosity using a Gaussian filter (sigma = 2.0 and support = 2.0) and an inverse threshold between −500.0 mgHA/cm^3^ and 261.8 mgHA/cm^3^ to select pore voxels. The apparent volume of the bleach samples was the TA times the length of the sample (as determined by calipers), not the apparent volume by Archimedes’ Principle.

### Statistical analysis

The Wilcoxon signed rank test was used to determine whether each bone property was significantly different between baseline and post‐manipulation (bleach experiment and ribose experiment). When there were multiple steps to the manipulation (baking experiment, boiling and pressure‐heating experiment, and glucose experiment), the Friedman test was used to determine whether the manipulation affected each bone property followed by Dunn's multiple comparisons test comparing each step to baseline (eg, baseline versus 4 weeks, baseline versus 8 weeks, and baseline versus 16 weeks within each glucose concentration or baseline versus each thermal denaturation temperature) if applicable. When baseline measurements were not available (eg, Raman measurements of samples incubated in PBS with or without sugar), the Friedman test was again used to match for donor followed by Dunn's multiple comparisons test (eg, 0.0 M sugar versus 0.1 M sugar, 0.0 M sugar versus 0.5 M sugar, and 0.1 M versus 0.5 M sugar) if applicable. Linear regression was used to relate post‐NaClO volume fraction of bound water to remaining organic fraction. All *p* values and adjusted *p* values from the aforementioned statistical tests were generated using GraphPad Prism software (version 6, La Jolla, CA, USA).

## Results

### Partial removal of the bone organic matrix decreased bound water content

**Figure 1:**
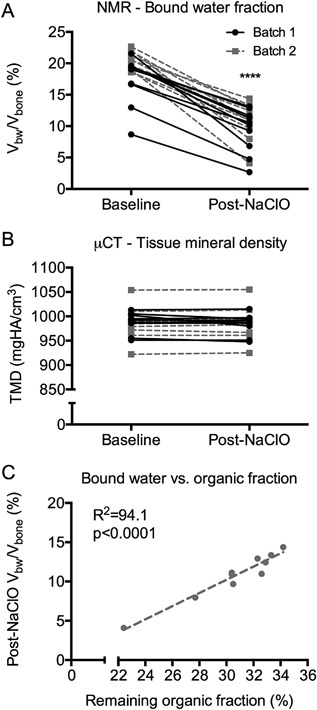
Effect of deproteinization on bound water fraction. After immersion of cortical bone for 96 hours in NaClO, the volume fraction of bound water decreased (*A*), while tissue mineral density did not change (*B*). For batch 2, the bound water fraction post‐NaClO treatment directly correlated with the remaining organic fraction as determined by gravimetric measurements (100 × (air‐dry mass − ash mass)/air‐dry mass) (*C*). A similar linear regression for batch 1 is provided in Supplemental Fig. S1. ****p < 0.0001 from Wilcoxon signed rank test.

**Figure 2:**
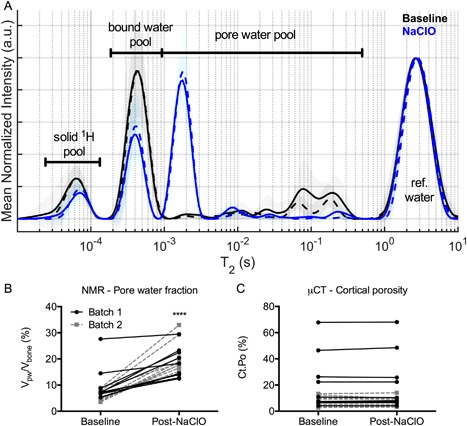
Effect of deproteinization on the T_2_ spectrum of bone (mean of each batch with SD depicted in the shading). Treatment of bone with NaClO for 96 hours decreased the bound water signals (baseline versus 96 hours: *p* < 0.0001) and shifted pore water signals from primarily long‐T_2_'s to short‐T_2_'s (*A*). This shift in T_2_'s translated to an increase in the volume fraction of pore water (*B*) after organic matter removal by NaClO, even though there was not an effect on cortical porosity as determined by µCT imaging (*C*). ****p < 0.0001 from Wilcoxon signed rank test.

Treatment of human cortical bone samples with sodium hypochlorite (NaClO) for 96 hours decreased the volume fraction of bound water (V_bw_/V_bone_) (Fig. 1 *A*) without any apparent effect on the mineral density (Fig. 1 *B*). There was still organic matrix present in the samples, and for batch 2, the remaining organic fraction (M_org_/M_dry_) strongly correlated with the post‐NaClO bound water fraction (Fig. 1 *C*). A similar correlation was also observed in batch 1 excluding one donor with high cortical porosity (>60%) such that there was more material lost than normal when transferring the dry specimen to the ashing crucible, resulting in a lower ash mass relative to other samples in batch 1 (Supplemental Fig. S1). The transverse relaxation time constant (T_2_) of the bound water pool decreased after treatment with NaClO (Fig. 2 *A*). Signals at longer T_2_'s (≃100 ms) in the pore water pool decreased, while signals at shorter T_2_'s (≃1 ms) in the same pool increased such that the overall volume fraction of pore water increased (Fig. 2 *B*) after bleaching, even though µCT‐derived cortical porosity did not change (Fig. 2 *C*).

### Denaturation of the bone collagen increased bound water content

**Figure 3:**
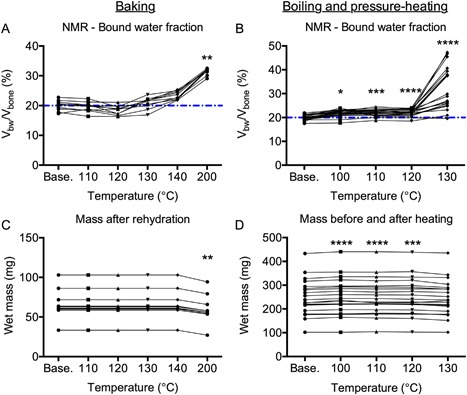
Effect of thermal denaturation of collagen on bound water fraction. Baking bone from 110°C to 140°C for 24 hours per temperature did not affect the volume fraction of bound water, while subsequently increasing the temperature to 200°C for 24 hours significantly increased bound water fraction relative to baseline (*A*). Boiling bone for 8 hours significantly increased bound water fraction, but the largest increase occurred after pressure‐heating the bone for 8 hours at 130°C (*B*). The dashed line is the median value for the baseline measurements. Rehydrating the bone samples for 24 hours kept the wet mass constant until baking at 200°C when possibly organic material was lost (*C*). Accompanying the small but significant increase in bound water fraction after boiling the bone samples was a small but significant increase in wet mass (*D*). *p < 0.05, **p < 0.005, ***p < 0.0005, and ****p < 0.0001 for temperature versus baseline from Dunn's multiple comparison test.

Progressively heating the bone caused an increase in the bound water fraction with respect to the baseline measurements. The largest change in the V_bw_/V_bone_ occurred at 200°C in the baking experiment (Fig. 3 *A*) and at 130°C in the boiling and then pressure‐heating experiment (Fig. 3 *B*). The wet mass of the baked samples was consistent even though the samples had to be rehydrated after each increase in temperature. One exception was a decrease in the wet mass after heating at 200°C for 24 hours, indicating that some organic matter was perhaps lost during this final thermal denaturing step (Fig. 3 *C*). Boiling the samples for 8 hours increased the wet mass, which may explain the increase in bound water fraction after boiling. The wet mass remained higher than the initial wet mass until the final 8 hours at 130°C (Fig. 3 *D*).

**Figure 4:**
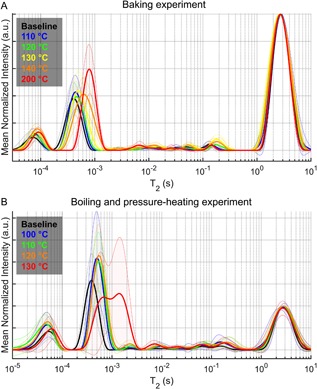
Effect of thermal denaturation of collagen on the T_2_ spectrum of bone (mean of each temperature with SD depicted in the shading). As the baking temperature increased, the T_2_ of the bound water pool shifted to higher values, while the distribution of T_2_ values within the pore water pool were seemingly unaffected (*A*). In the boiling and pressure‐heating experiment, the signals of the bound water pool shifted to higher T_2_ values, while the intensity of pore water pool signals at short‐T_2_'s (>1 ms) increased (*B*). At the highest temperature, the bound and pore water pools mixed.

Regardless of the method used to thermally denature collagen, the T_2_ of the bound water pool increased (slower relaxation rate) as the heating temperature increased (Tables 1 and 2 and Fig. 4 *A*, *B*). Interestingly, the shift in T_2_ at 130°C (boiling and pressure‐heating experiment) was large enough that the bound water signals mixed with the short‐T_2_ signals of the pore water pool (Fig. 4 *B*), but the degree to which mixing occurred depended on individual donor (Supplemental Fig. S2). Upon removing the data from the 9 donors in which the majority of the apparent bound water signal relaxed with T_2_'s >1 ms after pressure heating at 130°C, the bound water fraction at the end of thermal treatment (25.3% ± 3.1%) was still higher than the bound water fraction at baseline before heating (19.6% ± 1.1%, adjusted *p* < 0.0001). The pore water fraction after rehydration did not differ from the baseline measurement in the baking experiment, though it was significantly lower after the bones were baked at 110°C and then rehydrated (Table 1). Along with the increase in wet mass after boiling (Fig. 3 *D*), the pore water fraction increased upon boiling and remained elevated at each subsequent pressure‐heating temperature (Table 2).

**Table 1:** Effect of Baking on Selected ^1^H NMR and RS Properties of Bone (Mean ± SD)

Property	Unit	Baseline	110°C	120°C	130°C	140°C	200°C
^1^H NMR							
Solid‐T_2_	µs	73.7 ± 14.2	76.1 ± 8.0	77.7 ± 9.9	81.8 ± 9.9	86.7 ± 7.5***	90.5 ± 5.6****
Bound‐T_2_	µs	410.5 ± 46.7	449.9 ± 42.9	472.3 ± 49.5	511.1 ± 59.8**	641.8 ± 86.4****	797.5 ± 62.5****
V_pw_/V_bone_	%	5.56 ± 2.05	3.96 ± 0.99*	4.55 ± 0.77	7.37 ± 1.25	6.64 ± 0.79	4.58 ± 0.81
Raman spectroscopy							
I_1667_/I_1640_	—	1.54 ± 0.04	1.57 ± 0.05	1.49 ± 0.05	1.51 ± 0.05	1.48 ± 0.04	1.73 ± 0.13**
I_1667_/I_1690_	—	1.55 ± 0.04	1.48 ± 0.05	1.40 ± 0.05**	1.39 ± 0.03**	1.39 ± 0.03***	1.35 ± 0.03****
Hyp/Pro	—	0.744 ± 0.021	0.762 ± 0.017	0.793 ± 0.035	0.841 ± 0.022**	0.910 ± 0.039****	0.972 ± 0.119****
CO_3_/ ν_1_PO_4_	—	0.196 ± 0.013	0.196 ± 0.007	0.190 ± 0.015	0.184 ± 0.012	0.179 ± 0.010*	0.182 ± 0.013*
1/FWHM[ν_1_PO_4_]	cm	0.0581 ± 0.0026	0.0590 ± 0.0020	0.0597 ± 0.0014	0.0604 ± 0.0012	0.0617 ± 0.0016***	0.0611 ± 0.0013*
ν_2_PO_4_/Amide III	—	1.35 ± 0.21	1.35 ± 0.17	1.47 ± 0.18	1.54 ± 0.19	1.75 ± 0.26**	2.74 ± 0.26****
ν_1_PO_4_/CH_2_‐wag	—	8.42 ± 1.34	8.81 ± 0.79	9.05 ± 1.03	9.08 ± 0.91	9.58 ± 0.72	13.02 ± 1.31*

^1^ H NMR = ^1^H nuclear magnetic resonance relaxometry; RS = Raman spectroscopy.

The samples (*n* = 10) were rehydrated before analysis, and after analysis, they were baked again for 24 hours at the next higher temperature (temperature versus baseline: *p < 0.05, **p < 0.005, ***p < 0.0005, ****p < 0.0001 baseline from Dunn's multiple comparison test).

**Table 2:** Effect of Boiling and Then Pressure‐Heating on Selected ^1^H NMR and RS Properties of Bone (Mean ± SD)

Property	Unit	Baseline	100°C	110°C	120°C	130°C
^1^H NMR						
Solid‐T_2_	µs	51.4 ± 7.3	46.8 ± 6.0	41.7 ± 6.6*	47.2 ± 6.7	61.6 ± 10.1
Bound‐T_2_	µs	384.1 ± 28.6	508.2 ± 29.4	542.4 ± 38.4*	567.9 ± 45.0*	598.5 ± 284.4****
V_pw_/V_bone_	%	3.06 ± 1.36	5.14 ± 1.12*	5.89 ± 1.26****	6.39 ± 1.78****	5.49 ± 1.31***
Raman spectroscopy						
I_1667_/I_1640_	—	1.56 ± 0.04	1.60 ± 0.04	1.63 ± 0.04**	Not analyzed	1.65 ± 0.03****
I_1667_/I_1690_	—	1.54 ± 0.03	1.55 ± 0.04	1.58 ± 0.04*	Not analyzed	1.59 ± 0.05**
Hyp/Pro	—	0.747 ± 0.016	0.747 ± 0.016	0.757 ± 0.030	Not analyzed	0.908 ± 0.1205****
CO_3_/ν_1_PO_4_	—	0.180 ± 0.007	0.162 ± 0.008*	0.154 ± 0.009****	Not analyzed	0.134 ± 0.010****
1/FWHM[ν_1_PO_4_]	cm	0.0593 ± 0.0012	0.0621 ± 0.0021**	0.0640 ± 0.0035****	Not analyzed	0.0690 ± 0.0034****
ν_2_PO_4_/Amide III	—	2.03 ± 0.13	2.04 ± 0.08	2.15 ± 0.15*	Not analyzed	3.80 ± 1.32****
ν_1_PO_4_/CH_2_‐wag	—	15.05 ± 1.04	15.12 ± 0.76	15.73 ± 0.97	Not analyzed	24.99 ± 8.65****

^1^ H NMR = ^1^H nuclear magnetic resonance relaxometry; RS = Raman spectroscopy.

The samples (*n* = 20) were boiled for 8 hours and then analyzed. Subsequently, they were sealed in Pyrex tubes of water and heated for 8 hours at ever higher temperatures (temperature versus baseline: *p < 0.05, **p < 0.005, ***p < 0.0005, ****p < 0.0001 from Dunn's multiple comparison test).

### Glycation of bone by ribose decreased bound water content

**Figure 5:**
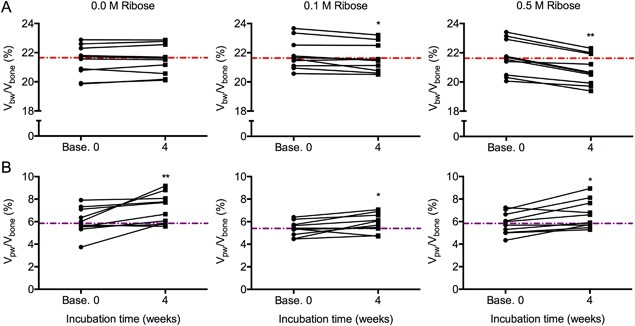
Effect of glycation by ribose on bound and pore water fractions. Incubating bone samples for 4 weeks in 0.1 M and 0.5 M ribose decreased the volume fraction of bound water, whereas control samples incubated in PBS did not experience a change in bound water fraction (*A*). Increase in the volume fraction of pore water was observed in all samples regardless of the concentration of ribose (*B*). The dashed lines are the median values of the baseline measurements per concentration. *p < 0.05 and **p < 0.005 from Wilcoxon signed rank test.

**Figure 6:**
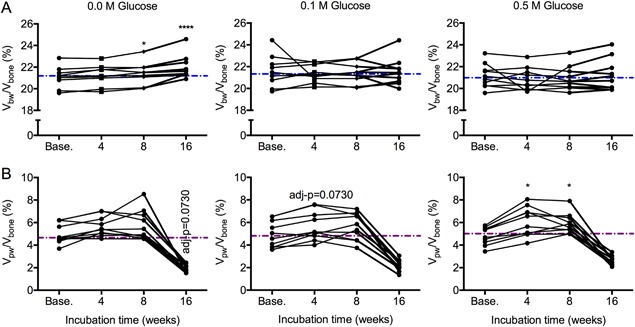
Effect of glycation by glucose on bound and pore water fractions. Incubating bone samples in glucose for 4 weeks to 16 weeks did not affect the volume fraction of bone water, while incubating bone in PBS for 8 weeks total increased bound water fraction (*A*). Regardless of the concentrator of glucose, the volume fraction of pore water tended to increase after 4 weeks and remained elevated after 8 weeks but then unexpectedly dropped after 16 weeks of incubation (*B*). The dashed lines are the median values of the baseline measurements per concentration. One donor in the 0.0 M glucose group with exceedingly high bound water after 8 weeks of incubation was removed from the graph (*A*) for clarity but not from the statistical analysis or the calculation of the median. *p < 0.05 and ****p < 0.0001 for incubation time versus baseline from Dunn's multiple comparison test; otherwise, *p* value provided.

Incubation of human cortical bone in 0.1 M or 0.5 M ribose at 37°C for 4 weeks significantly decreased the volume fraction of bound water, while incubation in control solution did not affect V_bw_/V_bone_ (Fig. 5 *A*). With the addition of pyridoxamine, there was still a significant decrease in bound water fraction in 0.1 M and 0.5 M ribose groups but not in the 0.0 M ribose group (Supplemental Fig. S3). Incubating bone in a less reactive sugar (0.1 M glucose and 0.5 M glucose) for a total of 16 weeks had no significant effect on bound water (Fig. 6 *A*). Unexpectedly, V_bw_/V_bone_ increased after 8 weeks in sodium phosphate buffer alone at 37°C (Fig. 6 *A*), even when an apparent outlier data point was removed (adjusted *p* = 0.0318 without it). Incubation of bone in sodium phosphate buffer increased the pore water fraction after 4 weeks or 8 weeks, regardless of the sugar concentration (Figs. 5 *B* and 6*B*), although this effect was less pronounced with the addition of pyridoxamine in the 4‐week ribose experiment (Supplemental Fig. S3). Glycation affected the T_2_ of the bound water pool, though the shift was modest (4‐week ribose in Supplemental Fig. S4 and 16‐week glucose in Supplemental Fig. S5). After incubation in PBS, the volume fraction of pore water did not vary much with different sugar concentrations with opposite trends observed in ribose versus glucose incubations (Tables 3 and 4), possibly due to differences in corresponding baselines (purple dashed lines in Fig. 5 *B* and Fig. 6 *B*), even though donors were matched across all groups. Thus, the presence of sugar likely did not affect pore water fraction of bone.

**Table 3:** The Effect of Glycation by Ribose on Selected ^1^H NMR and RS Properties of Bone (Mean ± SD)

Property	Unit	0.0 M Ribose	0.1 M Ribose	0.5 M Ribose	Friedman *p* value
^1^H NMR					
Solid‐T_2_	µs	38.2 ± 5.5	41.4 ± 6.5	39.6 ± 4.7	0.549
Bound‐T_2_	µs	448.0 ± 36.2	437.0 ± 28.6	400.5 ± 29.9^a^	0.002
V_pw_/V_bone_	%	7.2 ± 1.3	5.9 ± 0.8a	6.6 ± 1.2	0.026
Raman spectroscopy					
I_1667_/I_1690_	—	1.62 ± 0.05	1.60 ± 0.05	1.61 ± 0.05	0.436
CO_3_/ν_1_PO_4_	—	0.189 ± 0.011	0.194 ± 0.011	0.211 ± 0.015^a^	0.002
1/FWHM[ν_1_PO_4_]	cm	0.0572 ± 0.0037	0.0572 ± 0.00230	0.0571 ± 0.0038	0.974
ν_2_PO_4_/Amide III	—	1.77 ± 0.17	1.78 ± 0.10	1.77 ± 0.13	0.710
ν_1_PO_4_/CH_2_‐wag	—	7.64 ± 1.02	7.40 ± 0.53	7.33 ± 0.98	0.601

^1^ H NMR = ^1^H nuclear magnetic resonance relaxometry; RS = Raman spectroscopy.

Three neighboring bone samples from each donor (*n* = 10) were incubated for 4 weeks at one of three different concentrations of ribose (*n* = 10/concentration).

^a^ *p* < 0.05 for 0.1 M ribose or 0.5 M ribose versus 0.0 M ribose from Dunn's multiple comparison test.

**Table 4:** The Effect of Glycation by Glucose on Selected ^1^H NMR and RS Properties of Bone (Mean ± SD)

Property	Unit	0.0 M Glucose	0.1 M Glucose	0.5 M Glucose	Friedman *p* value
^1^H NMR					
Solid‐T_2_	µs	35.4 ± 5.6	35.7 ± 6.9	43.8 ± 19.8	0.164
Bound‐T_2_	µs	484.3 ± 69.8	458.1 ± 49.3^a^	442.6 ± 59.9a	0.008
V_pw_/V_bone_	%	1.9 ± 0.3	2.2 ± 0.5	2.6 ± 0.4^a^	0.001
V_bw_/V_bone_	%	22.3 ± 1.4	21.5 ± 1.2	21.2 ± 1.4^a^	0.001
Raman spectroscopy					
I_1667_/I_1640_	—	1.63 ± 0.02	1.62 ± 0.02	1.60 ± 0.01a	0.001
I_1667_/I_1690_	—	1.54 ± 0.04	1.56 ± 0.03	1.54 ± 0.06	0.601
Hyp/Pro	—	0.799 ± 0.009	0.804 ± 0.015	0.815 ± 0.014	0.078
CO_3_/ν_1_PO_4_	—	0.144 ± 0.009	0.149 ± 0.015	0.144 ± 0.006	0.319
1/FWHM[ν_1_PO_4_]	cm	0.0640 ± 0.0020	0.0638 ± 0.0013	0.0639 ± 0.0014	0.368
ν_2_PO_4_/Amide III	—	2.17 ± 0.11	2.12 ± 0.06	2.17 ± 0.12	0.974
ν_1_PO_4_/CH_2_‐wag	—	16.60 ± 1.37	16.03 ± 0.72	16.57 ± 1.28	0.135

^1^ H NMR = ^1^H nuclear magnetic resonance relaxometry; RS = Raman spectroscopy.

Three neighboring bone samples from each donor (*n* = 10) were incubated for 16 weeks at one of three different concentrations of glucose (*n* = 10/concentration).

^a^ *p* < 0.05 for 0.1 ribose or 0.5 M ribose versus 0.0 M ribose from Dunn's multiple comparison test.

### Thermal‐related changes in bound water fraction coincided with changes in the secondary structure of collagen I

To ascertain whether the thermal denaturing protocols affected the structure of organic matrix, we acquired Raman spectra from each sample before and after each heating step (except 120°C in the boiling and pressure‐heating experiment). As assessed by Amide I subpeak ratios that reflect the triple helical structure of collagen I,^31^, ^32^ a significant increase in the I_1667_/I_1640_ ratio occurred at 200°C (baking experiment) and 110°C (boiling and pressure‐heating experiment) (Table 1). Thermal treatment decreased and increased I_1667_/I_1690_ starting at 120°C (baking experiment) and at 110°C (boiling and pressure‐heating experiment), respectively (Table 2). Regardless of the thermal denaturing protocol, the hyroxyproline‐to‐proline ratio (Hyp/Pro) increased relative to baseline at 130°C (Tables 1 and 2). The largest changes in I_1667_/I_1640_ and in Hyp/Pro (Table 1 and Table 2) coincided with the largest changes in V_bw_/V_bone_ (Fig. 3). There were also apparent thermal effects on the mineral phase with both type B carbonate and crystallinity decreasing relative to baseline (Tables 1 and 2). There was a large increase in the mineral‐to‐matrix ratio at the highest temperature for each experiment, suggesting a loss of some organic matrix. Interestingly, the bound water fraction still increased with thermal denaturation and loss of the organic matrix with the largest increase occurring at 200°C or at 130°C (Fig. 3), namely the respective temperatures at which the largest increase in I_1667_/I_1640_ and in Hyp/Pro occurred (Tables 3 and 4).

### Glycation‐related differences in bound water fraction coincide with differences in the secondary structure of collagen I

**Figure 7:**
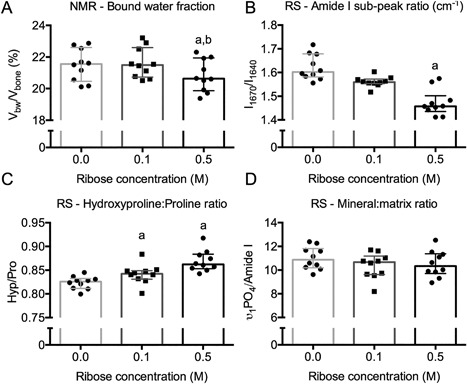
Ribose‐related differences in bound water fraction and secondary structure of collagen I of bone. After 4 weeks of incubation, the bound water fraction was lower for 0.5 M ribose than for 0.0 M and 0.1 M ribose groups (*A*). This difference coincided with a lower Amide I subpeak ratio for the 0.5 M ribose than for the 0.0 M ribose group (*B*). Compared with the control group, the hydroxyproline‐to‐proline ratio was higher with incubation in PBS with ribose (*C*), while there was not ribose‐related difference in the mineral‐to‐matrix ratio (*D*). ^a^ *p *< 0.05 for group versus 0.0 ribose and ^b^ *p* < 0.05 for group versus 0.5 M ribose from Dunn's multiple comparison test.

We measured Raman spectroscopy (RS) properties in control samples and in samples incubated in 0.1 M or 0.5 M sugar. In the 0.5 M ribose group, the lower bound water fraction compared with the control group (Fig. 7 *A*) was accompanied by a lower Amide I subpeak ratio (Fig. 7 *B*). Similarly, bound water fraction and I_1667_/I_1640_ ratio were lower in the 0.5 glucose group compared with the control group after 16 weeks of incubation (Table 4). The Hyp/Pro ratio was surprisingly higher for the glycated bones, regardless of concentration, than the nonglycated bones (Fig. 7 *C*), though differences in Hyp/Pro among the glucose groups did not reach statistical significance (Table 4). Incubating bone in sugar did not affect the mineral‐to‐matrix ratios (Fig. 7 *D*) or the mineral parameters (Tables 3 and 4). As the only exception, type B carbonate substitution was significantly higher in 0.5 M ribose samples compared with controls (Table 3). This was not the case for the glucose experiment (Table 4).

## Discussion

A primary determinant of bound water fraction of bone, as measured by ^1^H NMR relaxometry, is the amount of organic material in the matrix. A decrease in organic matrix caused a decrease in the volume fraction of bound water of cadaveric bone, and moreover, the remaining weight fraction of organic material directly correlated with the volume fraction of bound water after treatment (Fig. 1). Therefore, the clinical measurement of bound water using UTE‐MRI is likely an assessment of the amount of hydrated organic matrix within a patient's bone, whereas X‐ray‐based imaging tools provide an assessment of the mineral content of the bone. Modification of bone matrix by sugars appears to be a modulator of matrix‐bound water as glycated bone had lower bound water fraction than nonglycated bone (Figs. 5 and 7 and Table 4). Although thermal denaturation of collagen can lower work‐to‐fracture of cortical bone,^26^ denaturation in the current study unexpectedly increased bound water fraction. However, rather high temperatures (above 140°C) or heating plus pressure (above 120°C) was required to cause a large increase in bound water fraction (Fig. 3). Although nonhelical collagen (ie, cleavable by α‐chymotrypsin) is present in bone,^15^, ^33^ its overall fraction relative to triple helical collagen may be small. Moreover, unlike AGEs, the content of nonhelical collagen in human bone does not appear to vary with age.^15^ Nonetheless, current assays that require cleavage of the peptide chains may underestimate the amount of nonhelical collagen in human bone, especially from elderly donors for which AGE accumulation could be significant. While MR‐derived bound water fraction is predominantly an indicator of the volume fraction or concentration of the hydrated organic matrix within bone, it is not necessarily less in brittle bone compared with tough bone.

Although the accumulation of bone mineral displaces bound water,^12^ the present study did not assess the relative contribution of type 1 collagen content and the degree of mineralization to the volume fraction of bound water in bone. Unfortunately, the corollary experiment to treatment by NaClO (ie, removing the mineral phase to increase bound water) is not meaningful when using ^1^H NMR relaxometry. Demineralizing bone with a chelating agent such as ethylenediaminetetraacetic acid (EDTA) would cause the bound and pore water pools to mix (ie, H_2_O can readily move between being free in a pore and being bound to the matrix), making them indistinguishable by T_2_ measurement. Regardless of whether differences in MR‐derived bound water fraction reflect differences in mineralization or differences in the organic matrix, bound water, to date, is positively related to mechanical properties of bone, including those dependent on the organic matrix.^3^, ^4^, ^5^, ^22^

The three different treatments of bone affected pore water and the transverse relaxation time constants of the various proton pools. Partial removal of organic matrix decreased long‐T_2_ signals while dramatically increasing short‐T_2_ signals within the pore water pool (Fig. 2 *A*). The loss of organic material likely opens channels between pores. These newly created pores would be very small as the diameter of collagen fibrils is 80 to 120 nm^34^ and presumably would connect with canalicular‐lacunar system (10 to 20 nm), which is connected to the vascular pores (50 to 200 µm). The water residing in these small pores relaxes faster (ie, has shorter T_2_) than the water residing in the larger vascular channels and in resorption sites.^35^ Also, the loss in long‐T_2_ signals could partially be related to a loss of fat after NaClO treatment as lipid signals overlap long T_2_ in the pore water pool.^25^

Another striking change in the T_2_ spectrum of proton signals in bone occurred after the thermal denaturation of type 1 collagen. With destabilization of the collagen I triple helix, both protons of the organic matrix and protons of the matrix‐bound water relaxed slower (Table 1). Given that the stability of collagen arises from hydrogen bonding within the triple helix (eg, between −NH^δ+^ and −OH^δ‐^), the unraveling of the triple helix breaks inner hydrogen bonds and possibly creates more space or sites for interactions with water, hence an increase in bound water fraction upon thermal denaturation and alterations in relaxation behavior of the solid and bound water pools. Heat treatment of the bone in this study was apparently sufficient not only to denature mineralized collagen fibrils but also to create cracks in the matrix, allowing bound and pore water pools to mix (Fig. 4 and Supplemental Fig. S2).

Incubation of bone at 37°C in sodium phosphate buffer for 4 or 8 weeks increased the signal intensities of pore water (Figs. 5 and 6). The change in V_pw_/V_bone_ was small and not always significant. Nonetheless, there is perhaps a simple explanation for why heating the bone can result in increased volume fraction of pore water. When hydrated bone was stored frozen, gas could be trapped in some pores. Even though bones were brought to room temperature and immersed in buffer before the baseline measurements, the gas bubbles could have remained trapped until the bone samples are incubated at physiological or higher temperatures for an extended period of time. The unexpected decrease in V_pw_/V_bone_ following the additional 8‐week period of incubation in glucose is difficult to explain. Suspecting that gas was being generated over time despite the incubation in buffered water (eg, decomposition of organic material within the bone), we air‐dried the samples and rehydrated them for 24 hours per step. This nearly returned V_pw_/V_bone_ to baseline values (Supplemental Fig. S6). At any rate, there appears to be a hydration phenomenon when incubating or boiling bone at elevated temperatures, but there is a limit to the number of times and to the period of time bone samples can be removed for NMR analysis and put back into incubation.

The RS analysis confirmed that the thermal treatment of bone affected the triple helical structure of collagen and that the changes in the Amide I subpeak ratio I_1670_/I_1640_ coincided with the increase in bound water fraction (Fig. 3 *A* and Table 1). This subpeak ratio is known to be sensitive to thermal denaturation I^32^ as well as fatigue.^36^ The other Amide I subpeak ratio, I_1670_/I_1690_, generally sensitive to changes in the secondary structure of collagen due to local irradiation at therapeutic doses (cross‐link or matrix maturity ratio defined by band fitting the Amide I peak at 1660 cm^−1^ and right shoulder at 1683 cm^−1^ 37) or disruption in enzymatic cross‐linking (same cross‐link or matrix maturity ratio^38^) significantly decreased after baking at 120°C (Table 1), a temperature that does not generally denature bone collagen^39^ and does not lower the toughness of bone.^26^ This ratio actually increased in the boiling and pressure‐heating experiment after 110°C (Table 2), suggesting that it is not simply a marker of helical structure. The Hyp/Pro ratio also significantly changed after thermal treatment for both experiments. Because enzymatic proline hydroxylation cannot take place in our ex vivo experiment, conformational changes in the collagen I triple helical structure is likely responsible for the observed changes in Hyp/Pro ratio. However, RS‐derived Hyp/Pro ratio is not a measurement of the capacity to collagen I to interact water via hydrogen bonding.

There were other interesting changes in the RS properties of thermally denatured bone, namely an increase in crystallinity and decrease in type B carbonate substitution. In another RS study involving thermal treatment, Gourrier and colleagues^40^ baked different 80 µm sections of bovine cortical bone for 1 hour at different temperatures. They too observed a strong background fluorescence for samples baked between 190°C and 210°C. However, in their study using peak area ratios, CO_3_/ν_1_PO_4_ did not vary among the temperatures. Also, while mineral‐to‐matrix ratios (eg, ν_1_PO_4_/CH_2_‐wag) increased relative to control with an increase in heating temperature, ν_1_PO_4_/CH_2_‐wag did not vary between 150°C and 210°C, whereas changes in our study occurred at the highest temperature. These discrepancies are likely due to difference in the time of heating (1 hour versus 24 hours in the present study), size of the samples, and our use of sequential heating that possibly caused a decrease in organic material. Also, differences in the post‐processing of the spectra could also affect the sensitivity to thermal‐related changes in RS properties of bone. Lastly, using X‐ray diffraction techniques, Gourrier and colleagues^40^ also found that the thickness of mineral crystals increased as the heating temperature increased, and this ultrastructural change may have translated to the higher crystallinity by RS observed in the present study. How such a change affects bound water is unknown.

Confirming our previous study of glycation effects on bone using a different Raman acquisition protocol,^30^ I_1670_/I_1640_ and Hyp/Pro ratios were the most sensitive to sugar‐mediated changes in bone (Fig. 7 and Table 3). As previously reported, glycation of bone by 0.5 M ribose (4 weeks) caused a much greater accumulation of AGEs than glycation of bone by 0.5 M glucose (16 weeks), hence the differential effect on bound water fraction. Of the selected Raman peak ratios, I_1670_/I_1640_ increased when bound water fraction increased (thermal denaturation experiment) and decreased when bound water fraction decreased (glycation experiment). Perhaps, the left shoulder of the Amide I band near 1640 cm^−1^ (note that the actual location varies) is indicative of the ability of collagen I to participate in hydrogen bonding with water. Lastly, we did not analyze the bones incubated in ribose and pyridoxamine by RS because the effect of ribose on V_bw_/V_bv_ was similar between the incubation solutions without pyridoxamine and the incubation solutions with pyridoxamine (Supplemental Fig. S3).

Clearly, our model systems including different bone treatments are limited in mimicking the in vivo processing of the bone organic matrix. In addition, freeze‐thaw cycles were necessary to practically complete the various evaluations, and these could also affect the matrix. Although pyridoxamine is a known inhibitor of AGEs, namely when glucose causes AGE accumulation,^41^, ^42^ we did not investigate whether a higher concentration of pyridoxamine (>50 mM) could prevent the ribose‐related decrease in bound water fraction over 4 weeks. A lower concentration of pyridoxamine (0.1 mM) was found to reduce the accumulation of fluorescent AGEs when human cortical bone was incubated in 0.1 M glucose for 7 days at 50°C.^43^ Because ribose is significantly more reactive than glucose and formation of ribose‐protein adducts (ie, AGE precursors) may be sufficient to affect bound water, it is possible that the PM concentration used in our study was not high enough to completely block the Maillard reaction between sugar and protein. Lastly, there are inherent limitations in the use of cadaver bone as donor information is limited. Thus, future preclinical and clinical in vivo studies are required to establish the relative contributions of 1) the amount of the organic matrix; 2) degree of mineralization and cross‐linking of the matrix; 3) assembly of collagen into a triple helix; and 4) presence of cells and fluid flow to the bound water and pore water content in bone. Clinical studies are also necessary to ascertain whether pathological changes to bound water and pore water (eg, due to osteoporosis) are similar to those observed following the selected in vitro manipulations of bone.

The partial removal of the organic matrix from human cortical bone significantly decreased the volume fraction of bound water and significantly increased the volume fraction of pore water, without affecting tissue mineral density and porosity. Bound water fraction was higher when bone collagen I was thermally denatured compared with native bone. Thermal denaturation of bone collagen I under pressure caused the proton pools of bound and pore water to mix. Glycation of bone decreased bound water fraction and had only small effects on pore water fraction. The highest changes in bound water coincided with highest changes in the secondary structure of collagen I, as assessed by Raman spectroscopy analysis of the Amide I band. The NMR‐derived measurement of bound water is not necessarily a direct surrogate of bone toughness because there are likely competing physical mechanisms affecting hydrogen bonding between water and the organic matrix. Nonetheless, this assessment of matrix‐bound water is sensitive to changes in the organic matrix representing a unique potential of bound water measurements in clinical setting using MR imaging.

## Disclosures

SU, MU, CJL, SA, MG, and PV do not have conflicts of interest to declare. JSN and MDD hold a patent for determining mechanical properties of bone T_2_‐derived measures.

## Supporting information

Supporting Table S1.

Supporting Figure S1.

Supporting Figure S2.

Supporting Figure S3.

Supporting Figure S4.

Supporting Figure S5.

Supporting Figure S6.
