# Supplementary material for: Manipulating the Amount and Structure of the Organic Matrix Affects the Water Compartments of Human Cortical Bone
Source: JBMR Plus. 2019 Jan 28;3(6):e10135. doi: 10.1002/jbm4.10135 (PMC6636778; doi:10.1002/jbm4.10135)
Supplement: Supplementary file 1 — Supporting Table S1. [file JBM4-3-na-s001.docx]

Supplemental Table 1. The age of each donor (in years) is given for the manipulation experiments

| Experiment | Females | Males |
| --- | --- | --- |
| Deproteinization with sodium hypochlorite - Batch 1 | 42, 55, 63, 87, 94 | 38, 81, 82, 97 |
| Deproteinization with sodium hypochlorite - Batch 2 | 32, 54, 60, 89 | 46, 57, 74, 81, 88 |
| Thermal denaturation by baking | none | 21, 31, 32, 33, 74, 81,  86, 89, 91, 98 |
| Thermal denaturation by boiling and then pressure heating | 29, 35, 42, 54, 55, 70, 87, 94, 94, 101 | 25, 33, 38, 53, 58, 74, 81, 82, 84, 91 |
| Ribose incubation and Ribose with pyridoxamine | 53, 54, 55, 57, 58 | 46, 53, 57, 58, 60 |
| Glucose incubation | 47, 53, 54, 57, 60 | 46, 53, 57, 58, 60 |
