# Supplementary figures and images for: Manipulating the Amount and Structure of the Organic Matrix Affects the Water Compartments of Human Cortical Bone
Source: JBMR Plus. 2019 Jan 28;3(6):e10135. doi: 10.1002/jbm4.10135 (PMC6636778; doi:10.1002/jbm4.10135)

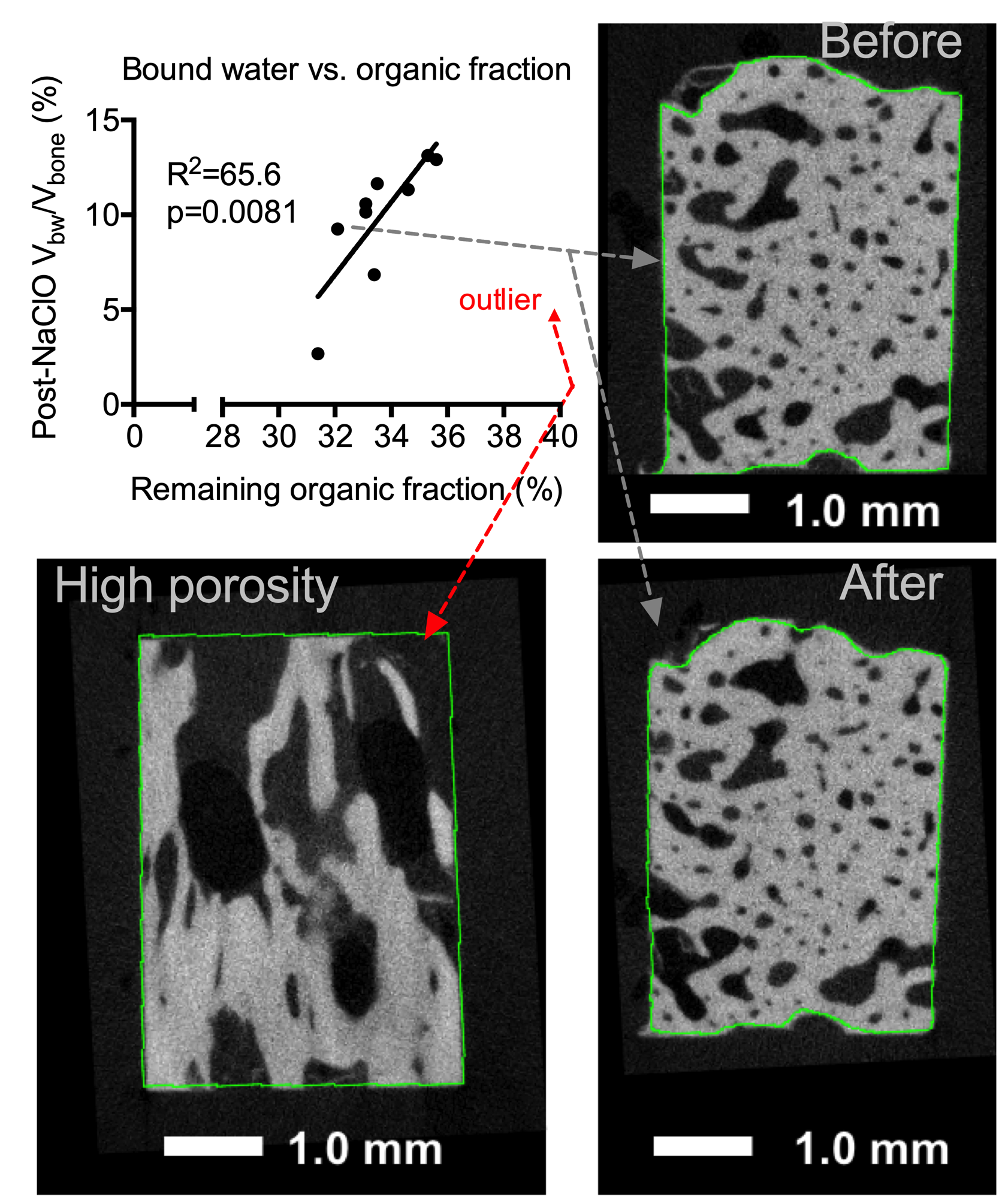

Supplement: Supplementary file 2 — Supporting Figure S1. [file JBM4-3-na-s002.tif]

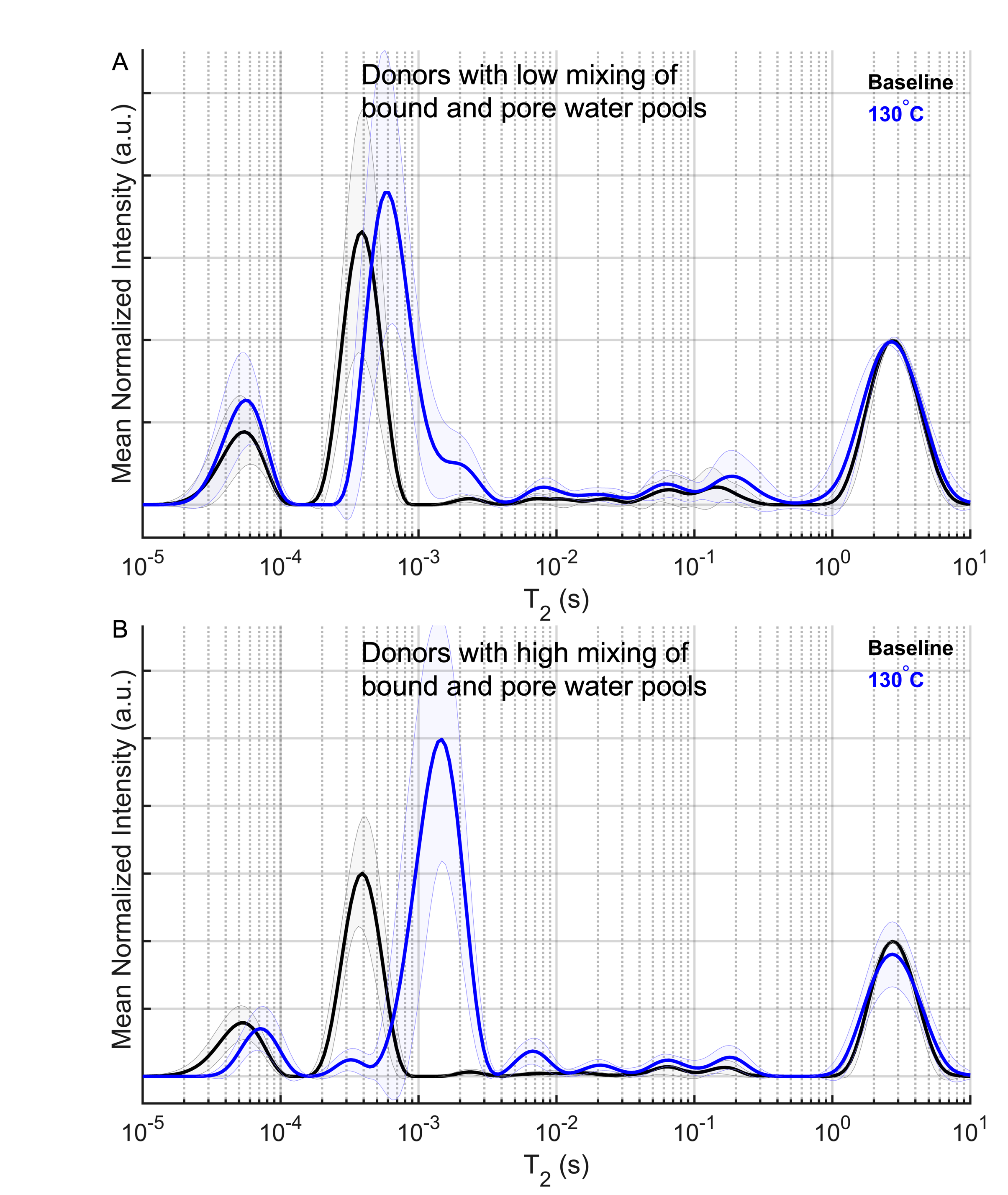

Supplement: Supplementary file 3 — Supporting Figure S2. [file JBM4-3-na-s003.tif]

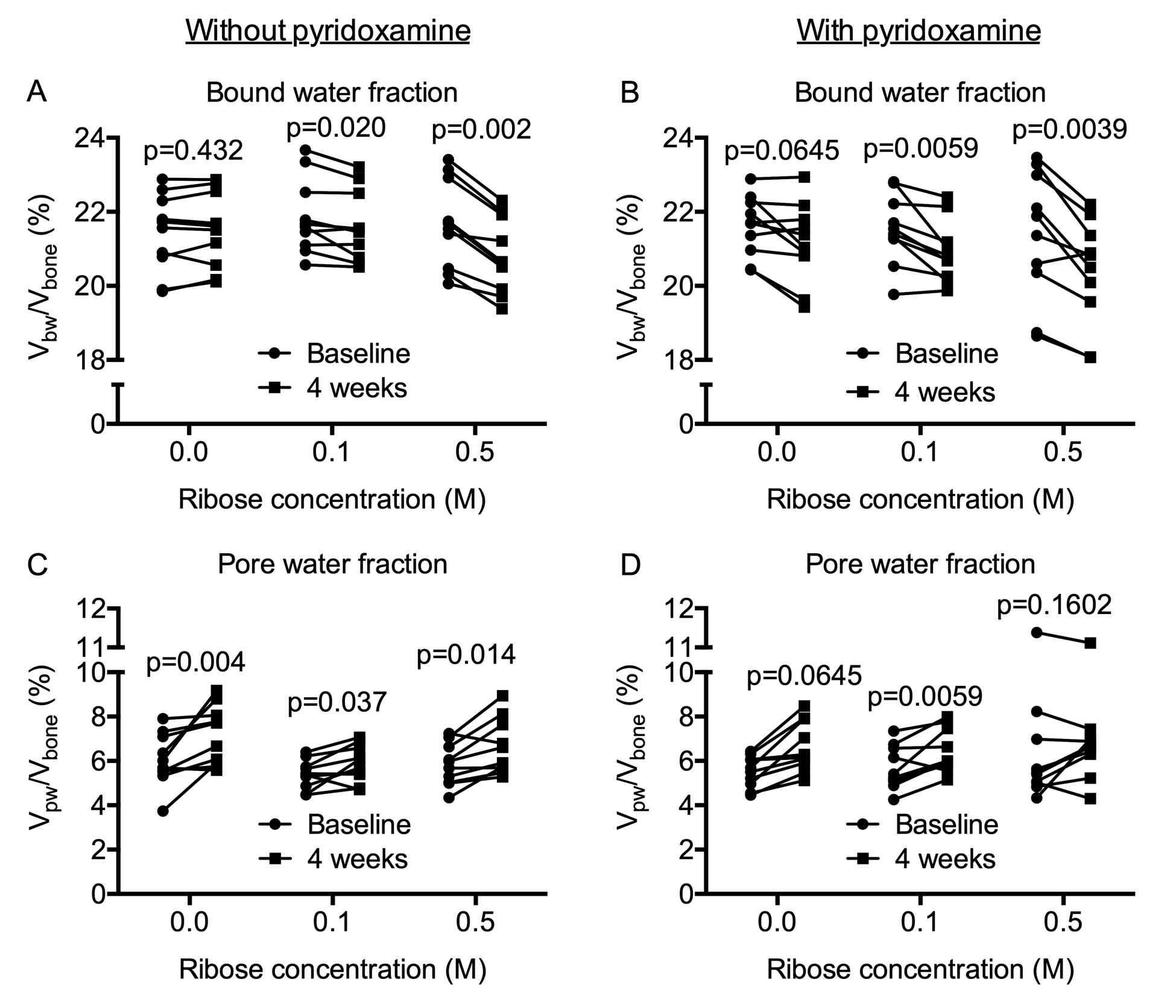

Supplement: Supplementary file 4 — Supporting Figure S3. [file JBM4-3-na-s004.tif]

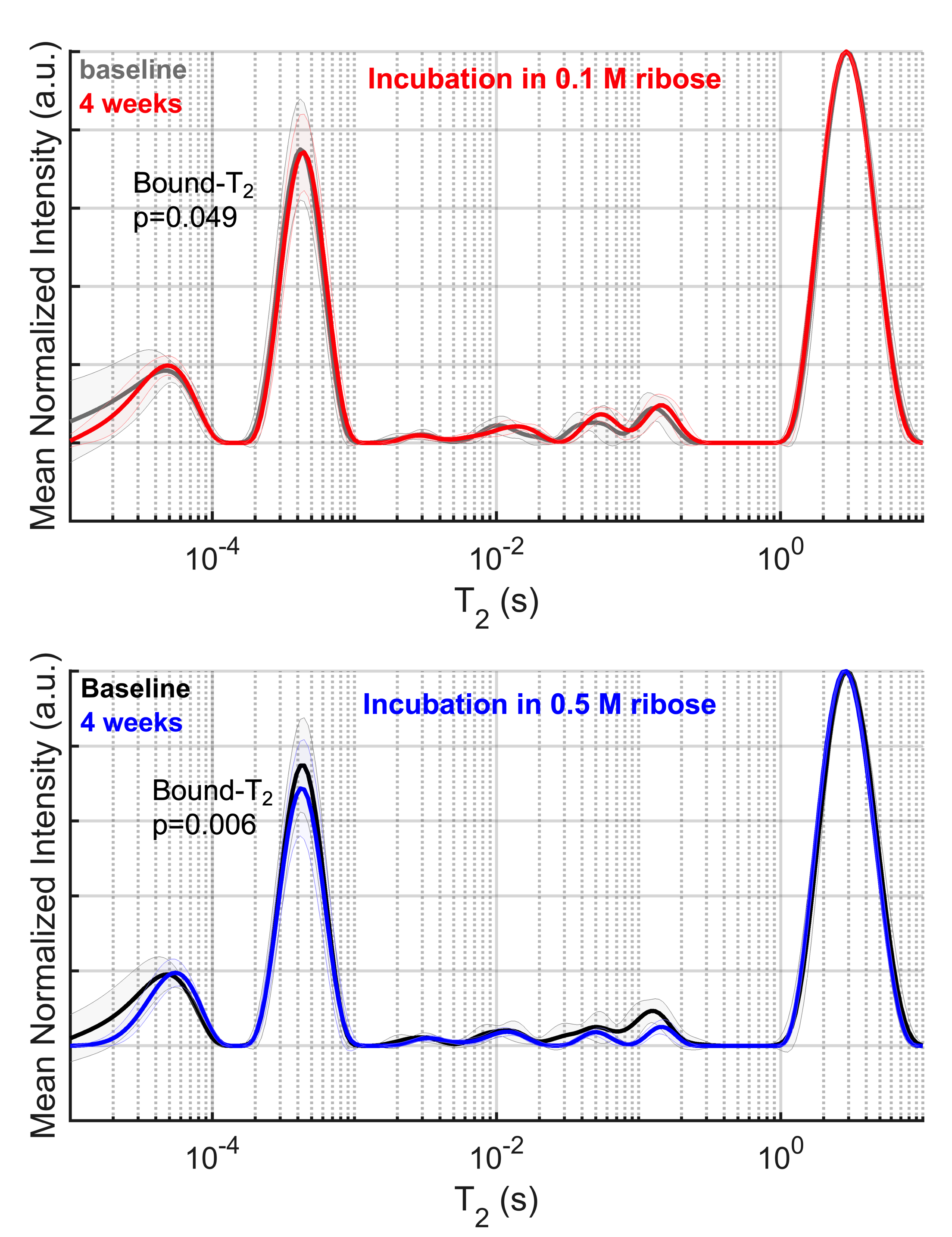

Supplement: Supplementary file 5 — Supporting Figure S4. [file JBM4-3-na-s005.tif]

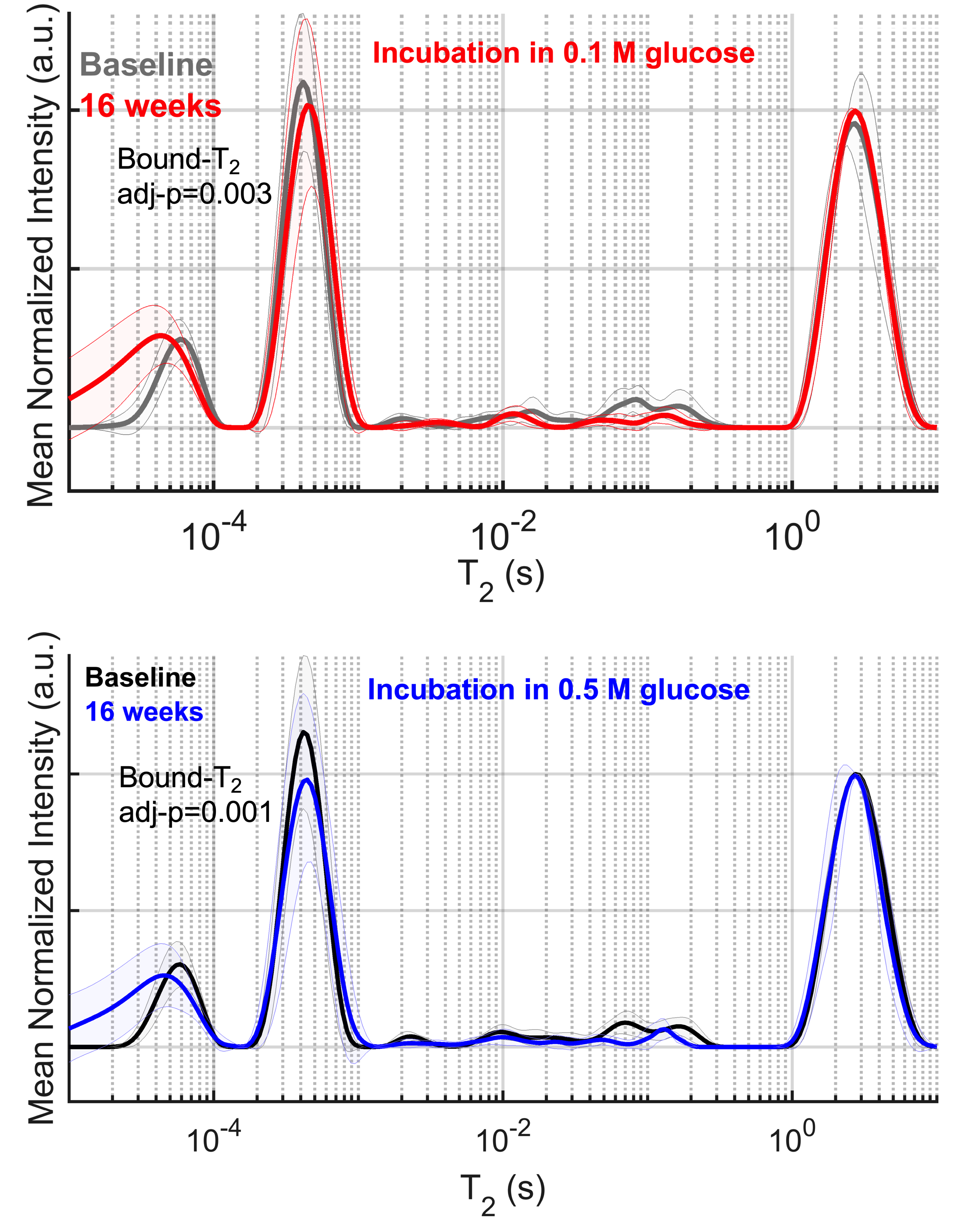

Supplement: Supplementary file 6 — Supporting Figure S5. [file JBM4-3-na-s006.tif]

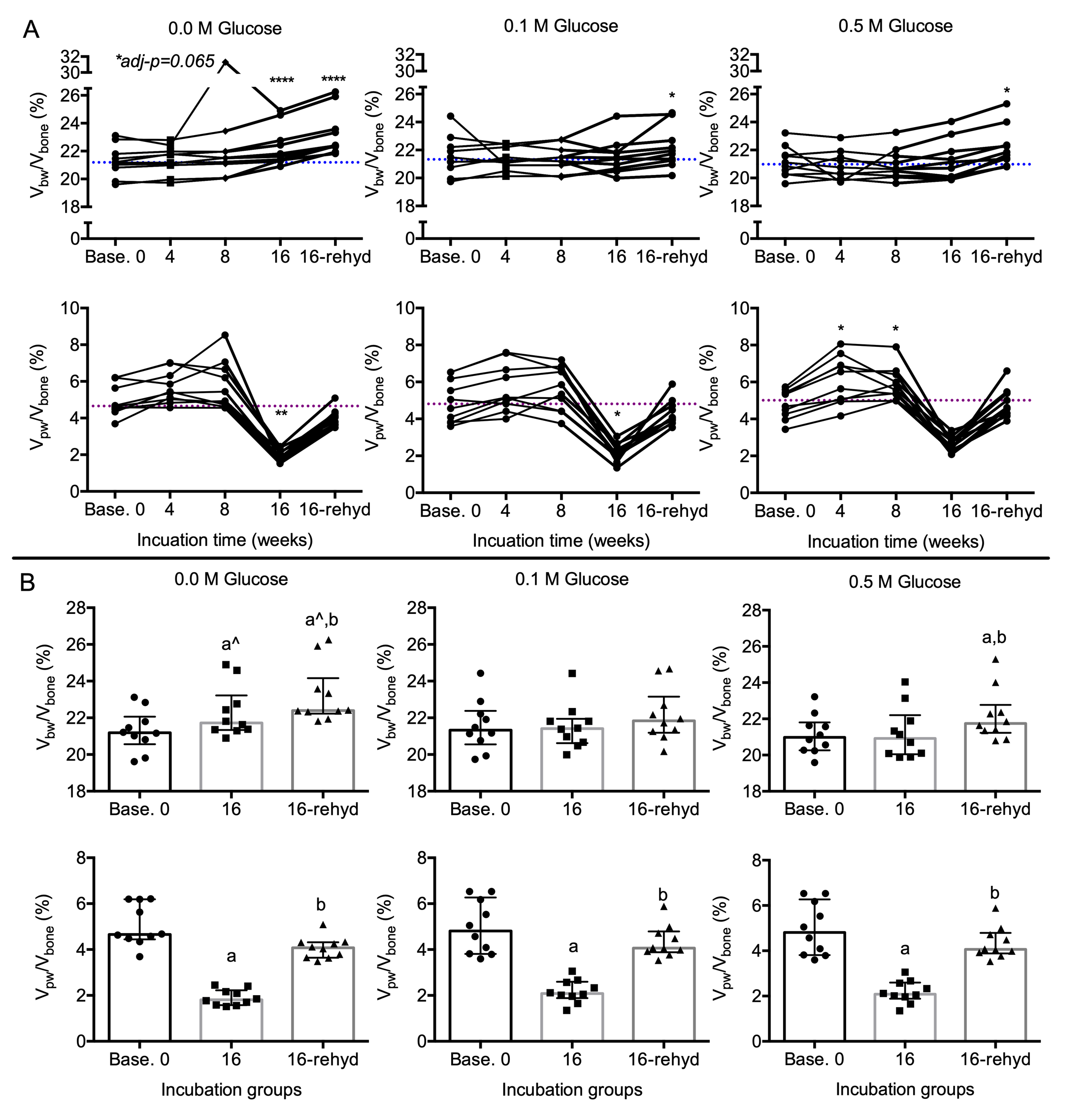

Supplement: Supplementary file 7 — Supporting Figure S6. [file JBM4-3-na-s007.tif]
